# Nutritional outcomes from a randomised investigation of intradialytic oral nutritional supplements in patients receiving haemodialysis, (NOURISH): a protocol for a pilot randomised controlled trial

**DOI:** 10.1186/2193-1801-2-515

**Published:** 2013-10-07

**Authors:** Louise Jackson, Benjamin Sully, Judith Cohen, Steven Julious

**Affiliations:** Dietetic Department, Sheffield Teaching Hospitals NHS Foundation Trust, Northern General Hospital, Herries Road, Sheffield, S5 7AU UK; School of Health and Related Research, University of Sheffield, 30 Regent Street, Sheffield, S1 4DA UK

**Keywords:** Oral nutritional supplement, Malnutrition, Renal, Haemodialysis, Handgrip strength

## Abstract

**Abstract:**

Haemodialysis is a form of renal replacement therapy but is a catabolic process that not only filters toxins but is also known to lead to amino acid losses. Patients with chronic kidney disease often have a poor appetite and this in combination with limited dietary intake and the detrimental effects of haemodialysis can lead to the development of malnutrition. Between 20% and 50% of haemodialysis patients are thought to be malnourished. Malnutrition can worsen clinical outcomes and increase the risk of hospitalisation. We hypothesise that a nutritional supplement taken during haemodialysis may help to improve nutritional status.

The aim of this study is to conduct a pilot randomised controlled trial to assess the use of an intradialytic nutritional supplement on nutritional status. The objectives are to assess the feasibility of the trial including: recruitment and retention of participants; preference of nutritional supplements; compliance with the intervention; ease of completion of the questionnaires and appropriateness of the tools used. Secondary outcomes include clinical outcomes to obtain variance in the patient population and estimates of effect size to inform the sample size for a future definitive trial.

The trial is a single centre, randomised, parallel-group, two armed external pilot with an intervention and control group. The intervention group will take a nutritional supplement each dialysis session from a choice of prescribable drink or pudding style supplements. The control group will receive standard care.

Recruitment and feasibility elements are the primary outcomes. Recruitment will be to time (*t* = 6 weeks). In order to collect sufficient data to inform a future sample size calculation, we will aim to recruit 30 participants to obtain 12 evaluable per arm anticipating some drop out. Secondary outcome measures include clinical variables; hand grip strength, quality of life, weight and biochemistry completed at baseline, 1 and 2 months.

Descriptive statistics will be used to analyse the baseline characteristics of the recruited participants. Means, confidence intervals and standard deviations will be reported for the outcome measures of handgrip strength, dietary intake, quality of life and weight.

**Trial registration:**

ISRCTN37431579.

## Background

Malnutrition can be described as a nutrient intake lower than the nutritional needs of the individual (Fouque et al. 2008a). The presence of malnutrition is reported in approximately 20 - 50% of the haemodialysis population (Kopple 1997; Marcen et al. 1997; Aparicio et al. 1999) and is a factor in protein and energy wasting (PEW). PEW can be defined as '’…a depletion of protein mass and / or energy fuel supplies…..” (Fouque et al. 2008a) and has many associated consequences including increased mortality and morbidity, decreased quality of life (QOL) and increased risk of hospitalisation. There are many causes of malnutrition in the haemodialysis population including a decreased appetite and subsequent oral intake due to uraemic toxins (Carrero et al. 2008), protein losses on dialysis (Wolfson et al. 1982) and the catabolic effect of dialysis (Kalantar-Zadeh et al. 2003; Ikizler 2007).

Interventions to improve nutritional status through an increase in nutrient intake include the encouragement of food fortification techniques through dietary counselling with a renal dietitian (Akpele and Bailey 2004; Fouque et al. 2007), the use of oral nutritional supplements (ONS) and enteral or parenteral nutrition (Stratton et al. 2005) all of which have been shown to improve markers of nutritional status in some way.

The measurement of nutritional status is particularly difficult due to its complex causes and multiple variables. Nutritional status cannot be measured with a single parameter but should be classified using a variety of reproducible measures that predict outcome (Fouque et al. 2007). In view of this, previous studies measuring the impact of ONS in haemodialysis patients have used a variety of measures including albumin (Sharma et al. 2002; Kalantar-Zadeh et al. 2005) subjective global assessment (Fouque et al. 2008b; Calegari et al. 2011), QOL (Scott et al. 2009; Calegari et al. 2011) and anthropometric measures such as tricep skinfold thickness, mid arm muscle circumference (Beutler et al. 1997) and handgrip strength (Leal et al. 2011).

### Rationale for pilot study

Many of the previous studies aiming to improve nutritional status in this population recommend the need for further research as the most appropriate type of nutritional support, the timing of ingestion and which nutritional markers should be used to best assess their efficacy are still not clear. Larger scale trials have not been conducted to date and there are a number of parameters which need investigation before a definitive protocol can be developed.

### Aim and objectives

The aim of this study is to conduct a randomised controlled external pilot trial to assess the effect of an intradialytic oral nutritional supplement on nutritional status.

The primary objectives are to: demonstrate the feasibility, practicality, safety and acceptability of a nutritional intervention study protocol, including the willingness to be randomised to treatment, in the haemodialysis population; To determine recruitment, withdrawal and drop-out rates and use these to estimate such parameters over the longer term; To identify any preference for types of nutritional supplement and adherence to the intervention schedule; To determine the completion rates of the study questionnaires; To assess QOL, exploring whether the frequency and length of the QOL assessment is acceptable to this patient population.

Secondary outcomes include clinical measures of weight, handgrip strength, biochemistry and QOL with the confounding factor of dietary intake also being assessed. These clinical parameters will be utilised to estimate the effect size and variability of outcome measures to inform the sample size calculation for a full trial.

## Methods/design

NOURISH is a two-arm parallel group randomised controlled external pilot trial of ONS versus standard care in haemodialysis patients. The trial will be conducted in a single centre with an intervention and control group. Both groups will receive standard care, but the intervention group will additionally take an ONS each dialysis session from a choice of prescribable drink or pudding style supplements in various flavours.

### Participant selection and setting

**Figure 1:**
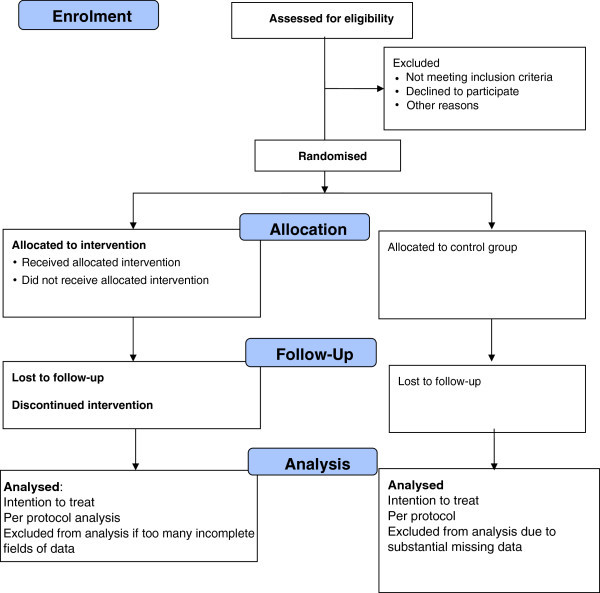
**CONSORT flowchart of trial.**

Adult renal haemodialysis patients will be recruited from an NHS hospital dialysis unit in Sheffield, UK. Patients will be identified, screened for eligibility and asked if they agree to participate by a renal dietitian when they attend one of their thrice weekly dialysis sessions. Inclusion and exclusion criteria are listed in Table 1. We will collect basic unidentifiable details on all eligible patients to allow completion of a CONSORT flow diagram (Figure 1).

**Table 1:** **Inclusion and exclusion criteria for study participants**

Inclusion criteria	Exclusion criteria
• Adult haemodialysis patients (≥18 years of age)	• Amputees
• Received dialysis for at least 6 months prior to study screening	• Significant oedema
• Receive haemodialysis at least 3 times per week at the main haemodialysis unit (not satellite centre)	• Non fluent English
• Body Mass Index ≤22 kg/m2	• Receiving nutritional supplementation prior to study commencing or within 1 month of commencement in the study
	• Participants with an allergy to any ingredients in the nutritional supplements
	• Persistent hyperkalaemia or hyperphosphataemia (defined as the last 3 months).

The inclusion criteria include a BMI ≤22 kg/m^2^ this was chosen as a BMI at the lower end of the normal range (18.5 -25 kg/m^2^) has been indicated as detrimental to haemodialysis patients (Fleischmann et al. 1999; Leavey et al. 2001).

Amputees and those with significant oedema or an unstable dry weight will be excluded from this pilot trial due to the inaccuracy of assessing the height and thus the BMI of amputees and the oedema / fluid overload of patients falsely elevating their weight and consequently their BMI.

### Sample size

To the authors knowledge no large randomised trials investigating the efficacy of intradialytic ONS have been conducted to date. Sufficient data on estimates of the standard deviation of the proposed outcome measures in this population are not available to enable a sample size to be calculated. This pilot trial is designed to collect this data to inform the sample size for a larger definitive trial in the future.

In order to collect sufficient data to inform a future sample size calculation we need 12 evaluable participants per arm, a total of 24 (Julious 2005). The aim will therefore be to recruit 30 patients to take into account any withdrawal.

### Trial treatment

All participants will continue to attend their usual haemodialysis sessions at a frequency of 3 times a week and may receive information from a renal dietitian as per standard treatment, regardless of group allocation. The intervention group will in addition, be requested to ingest 1 ONS per dialysis session over a 2 month intervention period. Participants will receive a choice of ONS; a drink or two styles of pudding in various flavours will be available, with the aim to improve adherence through choice. The supplements vary in their nutritional composition but will all provide between 200 and 300 Kcal and 10.5 – 12 g protein per dialysis session. The differences in composition are small but the specific supplements have been chosen due to the low fluid volume they provide, an important factor in an often fluid restricted population.

Participants will complete a short questionnaire each dialysis session regarding their general wellbeing, intradialytic oral intake and for those in the intervention arm information about the amount of supplement taken and any preferences for type of ONS.

Handgrip strength, measured by dynamometry, QOL measured using SF-12V2 (2012), Weight in kg, biochemical markers, and dietary intake will be measured at baseline, month 1 and at the end of the intervention (month 2).

These clinical endpoints will help determine the most appropriate outcome measures and timing of data collection points for a larger randomised controlled trial and inform the sample size calculation.

### Primary outcomes

This trial is designed to assess feasibility outcomes. The recruitment rate, calculated as the proportion of those screened who are subsequently consented into the trial within the specified recruitment period will be assessed. The practicalities of recruitment and the study protocol including barriers to recruitment, adherence to the intervention (including palatability and preference for types of ONS), withdrawal and loss to follow-up, the feasibility, acceptability and appropriateness of data collection methods and data completion rates will also be recorded.

### Analysis

Primary analysis will be intention to treat but per protocol analysis will also be performed if relevant. Logs of patients screened and approached to participate, will be kept along with the time taken to approach, request consent and to complete each assessment to inform possible recruitment rates for future larger studies.

Data from all randomised patients will be analysed including those that may have withdrawn from the treatment arm but allow their data to be used for analysis up to the point of withdrawal. Descriptive statistics will be used to analyse the baseline characteristics of the recruited participants. Means, confidence intervals, minimum and maximum along with standard deviations will be reported for the secondary outcome measures of handgrip strength, dietary intake, QOL, biochemistry and weight.

### Ethical approval and research governance

The trial has approval from the Leeds East National Research Ethics Service Committee, reference number 13/YH/0092. The trial will be conducted in compliance with the protocol, GCP and regulatory requirements. The trial is sponsored by the local NHS Trust (Sheffield Teaching Hospitals NHS Foundation Trust) and will be subject to the research and development department management and monitoring procedures.

## Discussion

### Randomisation and blinding

Once eligibility has been confirmed and consent acquired the participants will be randomly allocated to treatment group via a bespoke web-based randomisation system provided by a University of Sheffield subsidiary company, EpiGenesys. The randomisation system will only reveal the allocated treatment (ONS or standard care) after participants details have been recorded and the participant entered into the trial. To reduce the risk of random imbalances in the number allocated to each arm of the trial, randomisation will be stratified by gender and age of participant. Due to the small sample size of this pilot study it may be difficult to stratify the sample population but we think it is important to test the randomisation system fully prior to a larger trial so stratification will be incorporated.

Due to the nature of the intervention trial participants and staff will not be blinded to treatment allocation. We will consider whether it would be better to include a placebo ONS in the definitive trial, but one of the aims of the pilot is to assess palatability of different styles of ONS so it is not feasible to blind in the pilot. The outcome assessor performing the handgrip strength and dietary assessment is also not blinded in the pilot but this will be addressed for the definitive trial.

Any side effects or adverse events reported by the patient, observed by clinical staff or determined during case note review will be recorded. There is a small risk of gastrointestinal disturbances and hypotension following ingestion of the ONS. There is a small risk attached to the conduction of the handgrip assessment, it could cause bleeding from the arteriovenous fistula, and so will be conducted on the arm without vascular access, this will also be documented to aid analysis.

### Trial status

Recruitment to the trial is underway at the time of manuscript submission. Recruitment is due to take place between May and June 2013. The collection of participant data will conclude in August 2013, and analysis is planned to be completed by December 2013.

## Authors’ information

LJ is a senior Renal Dietitian by profession, currently completing an MSc in Clinical Research at the University of Sheffield and therefore plays a key role in the proposal of the pilot trial to inform in a key clinical area.

JC is a Research Fellow at the University of Sheffield Clinical Trials Research Unit, which is part of the School of Health and Related Research. JC is the supervising academic overseeing the research.

SJ is Professor of Medical Statistics at the University of Sheffield School of Health and Related Research. BS is a Trainee Medical Statistician within the department.

## Abbreviations

ONS: Oral nutritional supplement
PEW: Protein and energy wasting
QOL: Quality of life.

## References

[CR1] Akpele L, Bailey JL (2004). Nutrition counseling impacts serum albumin levels. J Ren Nutr.

[CR2] Aparicio M, Chauveau P, Azar R, Canaud B, Laville M, Leverve X, Group S, Herriot E (1999). Nutritional status of haemodialysis patients: a French national cooperative study. Nephrol Dial Transplant.

[CR3] Beutler KT, Park GK, Wilkowski MJ (1997). Effect of oral supplementation on nutrition indicators in hemodialysis patients. J Ren Nutr.

[CR4] Calegari A, Barros EG, Veronese FV, Thome FS (2011). Malnourished patients on hemodialysis improve after receiving a nutritional intervention. J Bras Nefrol.

[CR5] Carrero JJ, Aguilera A, Stenvinkel P, Gil F, Selgas R, Lindholm B (2008). Appetite disorders in uremia. J Ren Nutr.

[CR6] Fleischmann E, Teal N, Dudley J, May W, Bower JD, Salahudeen AK (1999). Influence of excess weight on mortality and hospital stay in 1346 hemodialysis patients. Kidney Int.

[CR7] Fouque D, Vennegoor M, ter Wee P, Wanner C, Basci A, Canaud B, Haage P, Konner K, Kooman J, Martin-Malo A, Pedrini L, Pizzarelli F, Tattersall J, Tordoir J, Vanholder R (2007). EBPG guideline on nutrition. Nephrol Dial Transplant.

[CR8] Fouque D, Kalantar-Zadeh K, Kopple J, Cano N, Chauveau P, Cuppari L, Franch H, Guarnieri G, Ikizler TA, Kaysen G, Lindholm B, Massy Z, Mitch W, Pineda E, Stenvinkel P, Treviño-Becerra A, Trevinho-Becerra A, Wanner C (2008a). A proposed nomenclature and diagnostic criteria for protein-energy wasting in acute and chronic kidney disease. Kidney Int.

[CR9] Fouque D, McKenzie J, de Mutsert RR, Azar R, Teta D, Plauth M, Cano N, Group RMTS (2008b). Use of a renal-specific oral supplement by haemodialysis patients with low protein intake does not increase the need for phosphate binders and may prevent a decline in nutritional status and quality of life. Nephrol Dial Transplant.

[CR10] Ikizler TA (2007). Protein and energy intake in advanced chronic kidney disease: how much is too much?. Seminars in dialysis.

[CR11] Julious SA (2005). Sample size of 12 per group rule of thumb for a pilot study. Pharm Stat.

[CR12] Kalantar-Zadeh K, Ikizler TAA, Block G, Avram MM, Kopple JD (2003). Malnutrition-inflammation complex syndrome in dialysis patients: causes and consequences. Am J Kidney Dis.

[CR13] Kalantar-Zadeh K, Braglia A, Chow J, Kwon O, Kuwae N, Colman S, Cockram DB, Kopple JD (2005). An anti-inflammatory and antioxidant nutritional supplement for hypoalbuminemic hemodialysis patients: a pilot/feasibility study. J Ren Nutr.

[CR14] Kopple JD (1997). Protein-energy malnutrition in maintenance dialsis patients. Am J Clin Nutr.

[CR15] Leal VO, Mafra D, Fouque D, Anjos LA (2011). Use of handgrip strength in the assessment of the muscle function of chronic kidney disease patients on dialysis: a systematic review. Nephrol Dial Transplant.

[CR16] Leavey SF, McCullough K, Hecking E, Goodkin D, Port FK, Young EW (2001). Body mass index and mortality in “healthier” as compared with “sicker” haemodialysis patients: results from the Dialysis Outcomes and Practice Patterns Study (DOPPS). Nephrol Dial Transplant.

[CR17] Marcen R, Teruel J, Angel de la Cal M, Gamez C (1997). The impact of malnutrition in morbidity and mortality in stable haemodialysis patients. Nephrol Dial Transplant.

[CR18] Scott MK, Shah NA, Vilay AM, Thomas J, Kraus MA, Mueller BA (2009). Effects of peridialytic oral supplements on nutritional status and quality of life in chronic hemodialysis patients. J Ren Nutr.

[CR19] (2012). Qualitymetric Incorporated.

[CR20] Sharma M, Rao M, Jacob S, Jacob CK (2002). A controlled trial of intermittent enteral nutrient supplementation in maintenance hemodialysis patients. J Ren Nutr.

[CR21] Stratton RJ, Bircher G, Fouque D, Stenvinkel P, de Mutsert R, Engfer M, Elia M (2005). Multinutrient oral supplements and tube feeding in maintenance dialysis: a systematic review and meta-analysis. Am J Kidney Dis.

[CR22] Wolfson M, Jones MR, Kopple JD (1982). Amino acid losses during hemodialysis with infusion of amino acids and glucose. Kidney Int.

